# Estrogen Regulates Protein Synthesis and Actin Polymerization in Hippocampal Neurons through Different Molecular Mechanisms

**DOI:** 10.3389/fendo.2014.00022

**Published:** 2014-02-25

**Authors:** Victor Briz, Michel Baudry

**Affiliations:** ^1^Graduate College of Biomedical Sciences, Western University of Health Sciences, Pomona, CA, USA

**Keywords:** estradiol, synaptic plasticity, protein synthesis, actin cytoskeleton, BDNF, calpain

## Abstract

Estrogen rapidly modulates hippocampal synaptic plasticity by activating selective membrane-associated receptors. Reorganization of the actin cytoskeleton and stimulation of mammalian target of rapamycin (mTOR)-mediated protein synthesis are two major events required for the consolidation of hippocampal long-term potentiation and memory. Estradiol regulates synaptic plasticity by interacting with both processes, but the underlying molecular mechanisms are not yet fully understood. Here, we used acute rat hippocampal slices to analyze the mechanisms underlying rapid changes in mTOR activity and actin polymerization elicited by estradiol. Estradiol-induced mTOR phosphorylation was preceded by rapid and transient activation of both extracellular signal-regulated kinase (ERK) and protein kinase B (Akt) and by phosphatase and tensin homolog (PTEN) degradation. These effects were prevented by calpain and ERK inhibitors. Estradiol-induced mTOR stimulation did not require activation of classical estrogen receptors (ER), as specific ERα and ERβ agonists (PPT and DPN, respectively) failed to mimic this effect, and ER antagonists could not block it. Estradiol rapidly activated both RhoA and p21-activated kinase (PAK). Furthermore, a specific inhibitor of RhoA kinase (ROCK), H1152, and a potent and specific PAK inhibitor, PF-3758309, blocked estradiol-induced cofilin phosphorylation and actin polymerization. ER antagonists also blocked these effects of estrogen. Consistently, both PPT and DPN stimulated PAK and cofilin phosphorylation as well as actin polymerization. Finally, the effects of estradiol on actin polymerization were insensitive to protein synthesis inhibitors, but its stimulation of mTOR activity was impaired by latrunculin A, a drug that disrupts actin filaments. Taken together, our results indicate that estradiol regulates local protein synthesis and cytoskeletal reorganization via different molecular mechanisms and signaling pathways.

## Introduction

A large amount of literature indicates that gonadal steroids have a strong impact on various functions and features of the central nervous system, such as the regulation of reproductive behavior and cognition as well as neuroprotection. The hippocampus is one of the main target regions for estrogen actions in brain. In particular, fluctuations of circulating levels of estrogen during the estrous cycle have been shown to modify spine density in area CA1 of hippocampus (1). These transient (over the course of 2–4 days) synaptic modifications are thought to be responsible for the facilitatory effects of estradiol on hippocampal long-term potentiation (LTP) (2), a mechanism widely considered to be involved in certain forms of learning and memory. In support of this notion, estrogen depletion in rats following ovariectomy is associated with cognitive decline and this deficit can be reversed by acute treatment with estradiol (3, 4). In addition, growing evidence indicates that estrogen causes rapid changes in neuronal excitability by activating membrane-associated estrogen receptors (5–8). Therefore, both genomic and non-genomic actions contribute to estrogen-mediated regulation of hippocampal synaptic plasticity. Yet, the molecular mechanisms and signaling pathways involved in this regulation are still poorly understood.

Among the physiological events participating in memory formation, reorganization of the actin cytoskeleton within dendritic spines is considered to be necessary for transforming a brief stimulus into a long-lasting memory trace (9, 10). Estradiol was recently reported to regulate actin cytoskeletal dynamics in hippocampus by stimulating the RhoA/RhoA kinase (ROCK) pathway (6), as it does in other cell types (11). Several studies have shown that estradiol can also activate Rac/p21-activated kinase (PAK) signaling in neurons providing another link between estrogen and the actin cytoskeleton (12–15). Inactivation of cofilin by LIM domain kinase 1 (LIMK1)-mediated phosphorylation at Ser 3 is a necessary step for estradiol-induced spine formation (16), and both ROCK and PAK1/4 can stimulate LIMK1 activity (17–19). However, it is not clear whether the Rac/PAK pathway contributes to the effects of estrogen on actin reorganization in hippocampus, and the specific contribution of these different cascades to estrogen-induced cofilin phosphorylation has not yet been tested under the same conditions.

Current hypothesis for LTP assumes a critical role for activity-induced release of brain derived neurotrophic factor (BDNF) and activation of its receptor, tropomyosin receptor kinase B (TrkB), resulting in stimulation of the mammalian target of rapamycin (mTOR) and local protein synthesis (20). Estrogen also stimulates dendritic protein synthesis in an extracellular signal-regulated kinase (ERK)- and protein kinase B (Akt)-dependent manner (21, 22). Furthermore, its pro-cognitive effects have been shown to rely on mTOR activity (23). Nevertheless, little is known regarding the precise molecular mechanism by which estradiol rapidly regulates protein translation in neurons. We recently identified a novel mechanism for BDNF-induced stimulation of mTOR-dependent dendritic protein synthesis, which involves calpain-2-mediated PTEN degradation (24). Since estradiol has been shown to activate calpain in dendritic spines through ERK-dependent phosphorylation (8), we sought to investigate whether estrogen could stimulate the same signaling cascade as BDNF. Our results indicate that estradiol-mediated mTOR activation in acute rat hippocampal slices is dependent on calpain-mediated PTEN truncation, ERK and TrkB receptor activities but independent of classical ERs. In contrast, stimulation of actin polymerization by estradiol requires activation of classical ERs as well as of ROCK and PAK signaling.

## Materials and Methods

Animals were treated in accordance with the principles and procedures of the National Institutes of Health Guide for the Care and Use of Laboratory Animals; all protocols were approved by the Institutional Animal Care and Use Committee of Western University of Health Sciences.

### Acute hippocampal slice preparation

Hippocampi were rapidly dissected from 2 to 3 month-old male Sprague-Dawley rats, transferred to oxygenated, ice-cold cutting medium containing (in millimolar): sucrose (220), NaCl (20), NaHCO_3_ (26), glucose (10), KCl (2.5), NaH_2_PO_4_ (1.25), MgSO_4_ (2), and cut into 400 μm thick transverse slices using a McIlwain tissue chopper. Hippocampal slices were maintained in a recovery chamber with artificial cerebrospinal fluid (aCSF) medium, containing (in millimolar): NaCl (124), KCl (2.5), CaCl_2_ (2.5), MgSO_4_ (1.5), NaH_2_PO_4_ (1.25), NaHCO_3_ (24), d-glucose (10), and saturated with 95%O_2_/5%CO_2_, for 1 h at 37°C. Hippocampal slices were then transferred into screw-cap microfuge tubes containing 3 ml of freshly oxygenated aCSF medium in the presence of various drugs.

### Cortical synaptoneurosome preparation

Synaptoneurosomes were prepared from 2- to 3-month-old male Sprague-Dawley rats, as previously described (25), with minor modifications. Briefly, cortices were rapidly dissected and placed into chilled modified Krebs solution (mKrebs) containing (in mM): NaCl (118.5), KCl (4.7), KH_2_PO_4_ (1.18), NaHCO_3_ (24.9), MgSO_4_ (1.18), CaCl_2_ (2.5), d-glucose (10), HEPES (1), pH 7.4, and equilibrated with 95%O_2_/5%CO_2_. Cortices were homogenized in a 7-ml Kontes tissue Dounce homogenizer with 10 strokes. Homogenized tissue was filtered through a 100-μm nylon mesh filter (BD Falcon) and the resulting suspension was filtered again through a 5-μm pore size Acrodisc syringe filter with a Supor membrane (Pall Life Sciences). The filtrate was then centrifuged at 1000 × *g* for 15 min at 4°C, washed once, and centrifuged again. The pellet was resuspended in mKrebs and various drugs were directly added to the pre-warmed (5 min, 37°C) synaptoneurosome suspension for the indicated periods of time.

### Drug treatments

Slices were incubated with 10 nM 17β-estradiol (Calbiochem) at 37°C for the indicated periods of times. Alternatively, slices were incubated with 100 ng/ml BDNF (Millipore), 100 nM 4,4′,4′′-(4-propyl-[1*H*]-pyrazole-1,3,5-triyl)*tris*phenol (PPT), 10 nM diarylpropionitrile (DPN), and 100 nM (±)-1-[(3aR*,4S*, 9bS*)-4-(6-bromo-1,3-benzodioxol-5-yl)-3a,4,5, 9b-tetrahydro- 3*H*-cyclopenta[*c*]quinolin-8-yl]-ethanone (G1) (all from Tocris Bioscience) for 30 or 60 min. We selected these concentrations for estrogen receptors (ERs) specific agonists based on previous reports showing a relatively low selectivity of DPN (about 30-fold) for ERβ over ERα, as compared to the much higher selectivity of PPT (around 1000-fold) for ERα over ERβ (26) and of G1 for G protein-coupled receptor 30 (GPR30) over ERα and ERβ (27). In some experiments, slices were pre-treated for 30 min with different inhibitors or antagonists, including U0126 (5 μM), K252a (1 μM), ICI 182780 (1 μM), 4-[2-phenyl-5,7-*bis*(trifluoromethyl)pyrazolo[1,5-*a*]pyrimidin-3-yl]phenol (PHTPP, 1 μM), cycloheximide (25 μM) (all from Tocris Bioscience), calpain-inhibitor 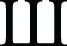 (10 μM, Calbiochem), (±)-(3aR*,4S*,9bS*)-4-(6-bromo-1,3-benzodioxol-5-yl)-3a,4,5,9b- tetrahydro-8-(1-methylethyl)-3*H*-cyclopenta[*c*]quinoline (G36, 1 μM, Azano Pharmaceuticals), H1152 (200 nM, Cayman chemicals), PF-3758309 (50–500 nM, ChemieTek), rapamycin (1 μM, Cell Signaling), and latruncullin A (0.5–5 μM, Sigma).

### Actin polymerization assay

Actin polymerization was quantified by measurement of “rhodamine-phalloidin fluorescent enhancement,” as previously described (28), with little modifications. In brief, hippocampal slices (three to six pooled slices) were washed twice with fresh aCSF after treatment, and subsequently fixed in PBS containing 4% paraformaldehyde and 1% octyl-β-d-glucopyranoside for 15 min at room temperature. After two rinses with PBS, slices were homogenized and centrifuged at 1000 × *g* for 1 min. Lysates were incubated with 15–30 nM phalloidin-tetramethylrhodamine B isothiocyanate (TRITC, Invitrogen) for 30–45 min at room temperature. After three washes, lysates were collected in 200 μl/slice of PBS and fluorescent intensity (excitation and emission wavelength were 546 and 590 nM, respectively) was determined using a POLARstar Omega fluorescence polarization microplate reader (BMG Labtech).

### RhoA activity assay

RhoA activity was determined by pull-down of RhoA-GTP with Rhotekin binding domain (RBD)-linked agarose beads (Millipore), as described previously (6). Rhotekin RBD binds strongly to both RhoA and RhoC, but weakly to RhoB (29). RhoC is not present in brain, thus RhoA was specifically detected in blots probed with RhoA antibody. Briefly, samples (6–10 pooled slices) were homogenized in Mg^2+^ lysis buffer (25 mM HEPES, pH 7.5, 150 mM NaCl, 1% Igepal CA-630, 10 mM MgCl_2_, 0.5 mM EDTA and 10% glycerol) containing a protease inhibitor Cocktail (Thermo Scientific). Protein levels were measured and equalized. Samples were incubated with Rhotekin RBD-agarose beads and gently rocked for 1 h at 4°C. Agarose beads were collected by centrifugation (30 s, 16000 × *g*, 4°C) and washed three times with Mg^2+^ lysis buffer, resuspended and boiled in Laemmli buffer for 5 min, and then separated by SDS-PAGE. Western blot analysis was performed using mouse anti-RhoA antibody (sc-418, Santa Cruz Biotechnology).

### Western blot

Protein lysates were prepared at a final concentration of 4–8 μg protein/μl by homogenization of rat hippocampal slices in ice-cold lysis buffer (50 mM Tris–HCl, pH 7.4, 1% Triton, 1 mM EDTA, 1 mM EGTA, 1 mM PMSF) containing a protease and phosphatase inhibitor cocktail (Thermo Scientific). After sample processing, 25–40 μg of denatured proteins were subjected to 6–15% SDS-PAGE and transferred onto polyvinylidene difluoride membranes. Membranes were blocked with 5% non-fat milk dissolved in Tris-buffered saline (TBS) for 30 min at room temperature and probed overnight with different primary antibodies at 4°C. Membranes were then washed three times with TBS containing 0.1% Tween 20 (TBST) for 10 min followed by incubation with Odyssey infrared-conjugated secondary antibodies diluted 1:10000 in Odyssey blocking buffer (LI-COR Biosciences) containing 0.1% Tween 20 and 0.05% SDS for 1 h at room temperature. After 3× 10 min washes with TBST, membranes were scanned using an Odyssey infrared imager (LI-COR Biosciences).

### Antibodies

All primary antibody solutions were prepared in TBST containing 5% bovine serum albumin at a 1:1000 dilution, except otherwise stated. The following primary antibodies were used: anti-phospho-mTOR (Ser2448) and anti-mTOR (both from Cell Signaling), anti-phospho-ERK1/2 (Thr202/Tyr204) and anti-ERK1/2 (both at 1:2000; Cell Signaling), anti-phospho-Akt (Ser473 and Thr308) and anti-Akt (all from Cell Signaling), anti-phospho-p70S6K (Thr389 from Millipore and Thr421/Ser424 from Cell Signaling), anti-p70S6K (Cell Signaling), anti-phospho-cofilin (Ser3, Abcam) and anti-cofilin (Cell Signaling), anti-phospho-PAK1/2/3 (Invitrogen) and anti-PAK3 (1:500, Millipore), anti-spectrin (1:2000; Millipore), anti-Arc (1:5000; Millipore), anti-actin (1:10000; Millipore), and anti-PTEN (Cell Signaling).

### Statistical analysis

Statistical comparisons were made by using one-way analyses of variance (ANOVA) followed by Dunnett’s multiple comparison test or two-way ANOVA followed by Bonferroni posttest analysis. Results were generally calculated as means ± standard error of the mean (SEM) from the indicated number of independent experiments and expressed as fold of the indicated control. Statistical significance was always referred to as: *p < 0.05, **p < 0.01, and ***p < 0.001, as compared to control (DMSO or time = 0 min); and ^#^*p* < 0.05, ^##^*p* < 0.01, and ^###^*p* < 0.001, as compared to BDNF or E2 alone; absence of symbol indicates *p* values >0.05, which were considered as not significant.

## Results

### Estradiol activates mTOR through the BDNF signaling pathway

**Figure 1:**
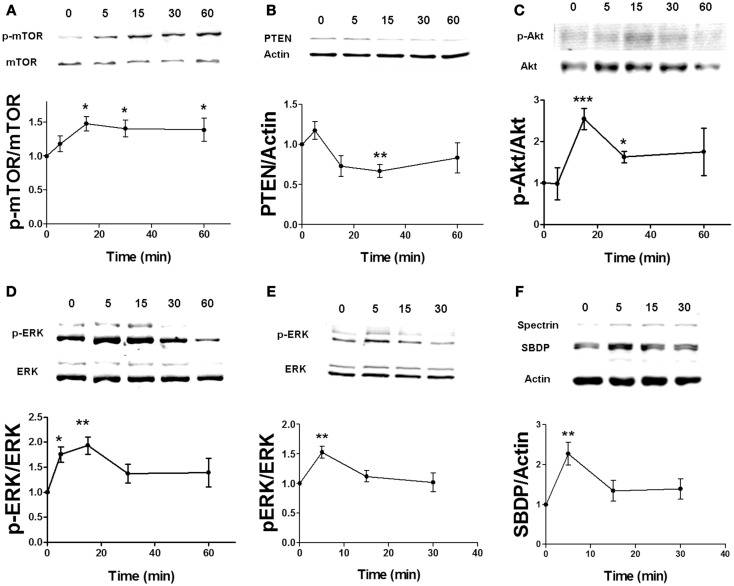
**Time course for estradiol-induced activation of mTOR signaling**. Acute hippocampal slices **(A–D)** or cortical synaptoneurosomes **(E,F)** were treated with estradiol (10 nM) for the indicated periods of times, and were homogenized and processed for western blot. Data are presented as the ratio (fold of control) of: **(A)** phospho-mTOR (p-mTOR) over total mTOR (*N* = 4–8), **(B)** PTEN over actin (*N* = 3–11), **(C)** phospho-Akt (p-Akt) over total Akt (*N* = 2–8), **(D)** phospho-ERK (p-ERK) over total ERK (*N* = 14–16), **(E)** p-ERK over total ERK (*N* = 4–5), **(F)** spectrin breakdown product (SBDP) over actin (*N* = 3–5); *p < 0.05, **p < 0.01, ***p < 0.001, as compared to control (0 min) (one-way ANOVA).

Based on our previous study on the regulation of the mTOR pathway by BDNF (24), stimulation of the BDNF receptor, TrkB, causes ERK-mediated calpain-2 activation, which leads to PTEN cleavage and subsequent mTOR phosphorylation via Akt stimulation. We first determined the time course of estradiol-mediated regulation of mTOR and of some of the signaling proteins upstream of mTOR in acute hippocampal slices. Both application of 10 nM estradiol increased mTOR phosphorylation within 15 min, and the effect persisted for at least 60 min (Figure 1A). Similarly, PTEN levels were decreased as early as 15 min and remained decreased for up to 1 h after estradiol treatment, although the effect was statistically significant only for the 30-min time point (Figure 1B). In contrast, activation of both ERK and Akt by estradiol was transient; ERK phosphorylation was increased within 5–15 min but not at later time points, whereas Akt activation (measured as increase in phospho-Akt levels at Ser473) peaked 15 min after estradiol application and gradually returned to baseline (Figures 1C,D). A similar time course was obtained for estradiol-induced phosphorylation of Akt at Thr308 (data not shown). We also assessed calpain activity in hippocampal slices by determining the levels of the calpain-specific spectrin breakdown product (SBDP) after estradiol treatment. However, we could not detect any changes in SBDP levels across the different time points, as compared to the control group (data not shown). This is likely due to the high basal levels of SBDP in hippocampal slices from adult rats, which has been previously documented (30). For this reason, we monitored the effects of estradiol on calpain activity using a different preparation, cortical synaptoneurosomes. We first verified that estradiol produced a similar pattern of ERK activation in synaptoneurosomes as in hippocampal slices. ERK phosphorylation was enhanced 5 min after estradiol treatment, but the effect returned to baseline levels faster (at 15 min) than in hippocampal slices (Figure 1E). Similarly, estradiol transiently increased the levels of SBDP in synaptoneurosomes (Figure 1F).

**Figure 2:**
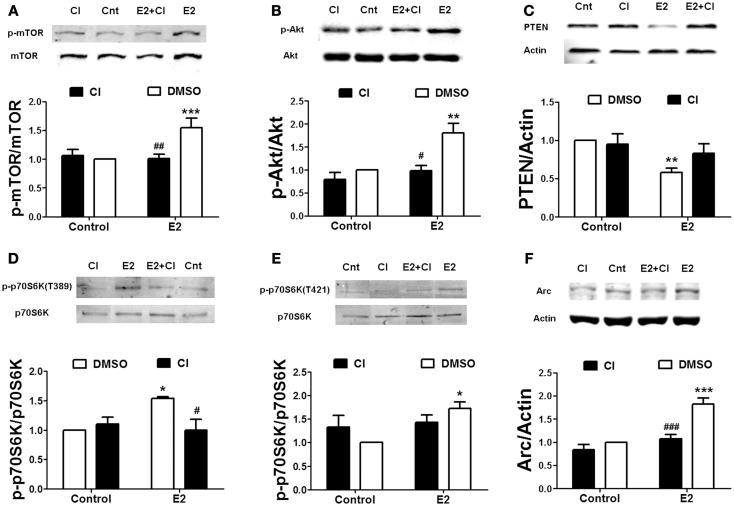
**Estradiol-induced activation of the mTOR pathway and protein synthesis requires calpain activation**. Acute hippocampal slices **(A–E)** or cortical synaptoneurosomes **(F)** were pre-treated with DMSO (Cnt) or calpain inhibitor III (CI, 10 μM) for 30 min and then incubated with estradiol (E2, 10 nM) for additional 30 min. At the end of treatments, samples were homogenized and processed for western blot. Data are presented as the ratio (fold of control) of: **(A)** p-mTOR over total mTOR (*N* = 3–11), **(B)** p-Akt over total Akt (*N* = 2–4), **(C)** PTEN over actin (*N* = 5–8), **(D)** p-p70S6K (T389) over total p70S6K (*N* = 3–4), **(E)** p-p70S6K (T421/S424) over total p70S6K (*N* = 5), **(F)** Arc over actin (*N* = 4–8); *p < 0.05, **p < 0.01, ***p < 0.001, as compared to control (DMSO); ^#^*p* < 0.05, ^##^*p* < 0.01, ^###^*p* < 0.001, as compared to E2 alone (two-way ANOVA).

To determine the link between estrogen-stimulated calpain activity and mTOR-dependent protein synthesis, we treated hippocampal slices with 10 μM calpain-inhibitor III (CI-III) before estradiol application and then examined mTOR activation. Calpain inhibition completely blocked estradiol-elicited increase in mTOR phosphorylation (Figure 2A). Pre-treatment with CI-III also prevented PTEN degradation and Akt activation caused by estradiol (Figures 2B,C). Similar results were obtained for estradiol-induced mTOR activation and PTEN degradation in cortical synaptoneurosomes (data not shown). One of the main targets of mTOR in neurons is the translation factor p70SK6, which is activated by phosphorylation at two different sites, Thr389 and Thr421/Ser424 (31). We then examined whether estradiol activates p70S6K in hippocampal slices. Treatment with estrogen enhanced the phosphorylation of p70SK6 at Thr389, and the effect was blocked by calpain inhibition (Figure 2D). In contrast, the increase in p70SK6 phosphorylation at Thr421/Ser424 caused by estradiol was not significantly inhibited by CI-III (Figure 2E), possibly because phosphorylation at these sites is not only mediated by mTOR but also by ERK (31). To further confirm that calpain activity was required for estradiol-mediated regulation of protein synthesis, we determined the effect of the hormone on the levels of Arc, a protein known to be rapidly up-regulated in response to synaptic activity (32). Estradiol significantly enhanced Arc levels in cortical synaptoneurosomes and the effect was blocked by CI-III pre-treatment (Figure 2F).

**Figure 3:**
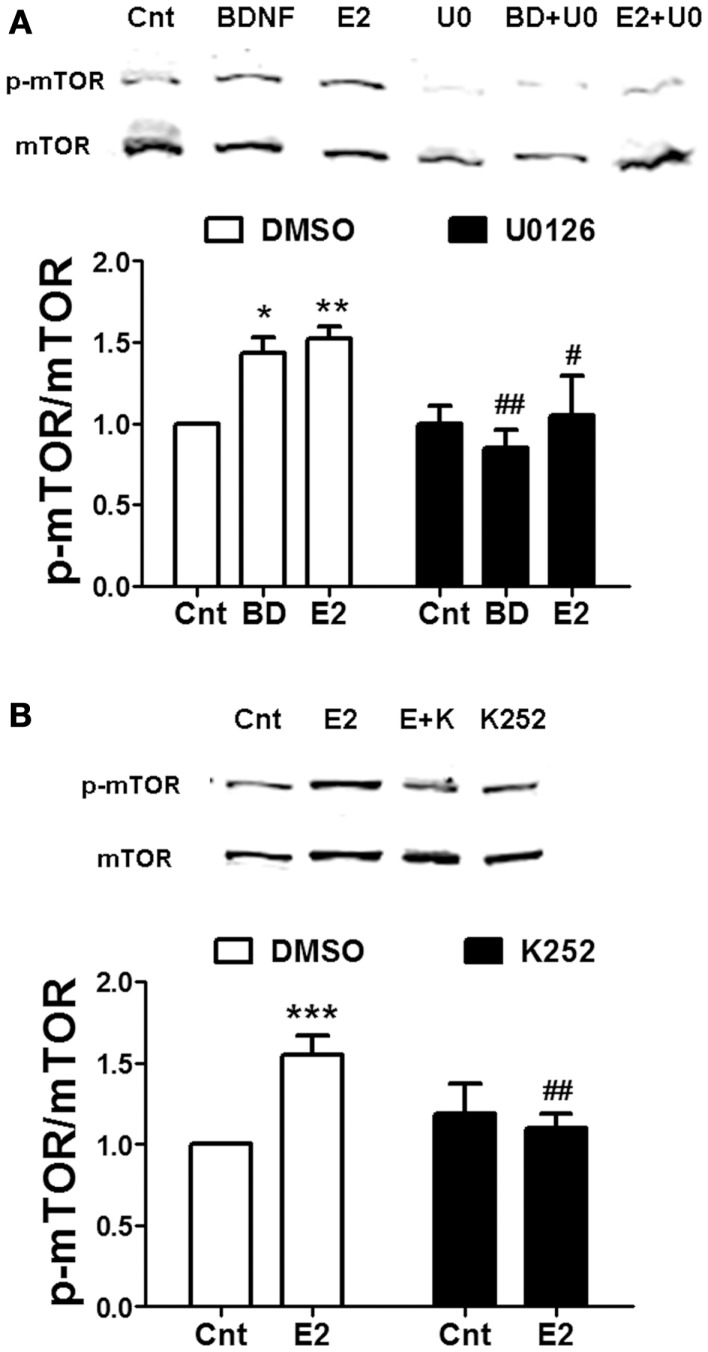
**Estradiol-induced mTOR phosphorylation involves ERK and TrkB receptor activation**. Acute hippocampal slices were pre-treated with DMSO (Cnt), U0126 (U0, 5 μM), or K252 (K, 1 μM) for 30 min and then incubated with estradiol (E2, 10 nM) or BDNF (BD, 100 ng/ml) for additional 30 min. At the end of treatments, samples were homogenized and processed for western blot. Data are presented as the ratio (fold of control) of: **(A)** p-mTOR over total mTOR (*N* = 3–9), **(B)** p-mTOR over total mTOR (*N* = 8–14); *p < 0.05, **p < 0.01, ***p < 0.001, as compared to control (DMSO); ^#^*p* < 0.05, ^##^*p* < 0.01, as compared to E2 or BDNF alone (two-way ANOVA).

Rapid calpain activation by estradiol and BDNF in dendritic spines is triggered by ERK-dependent phosphorylation (8, 28). If calpain activity is required for mTOR-mediated protein translation, ERK should be upstream of mTOR as well. When hippocampal slices were pre-treated with the ERK kinase specific inhibitor U0126 (5 μM), both estrogen- or BDNF-induced mTOR activation was prevented (Figure 3A). Since estradiol and BDNF were able to activate the same signaling cascade, we then tested the hypothesis that BDNF could mediate the effects of estradiol on mTOR activity. Pre-incubation of hippocampal slices with the TrkB receptor antagonist K252a (1 μM) blocked the increase in mTOR phosphorylation caused by estradiol (Figure 3B).

**Figure 4:**
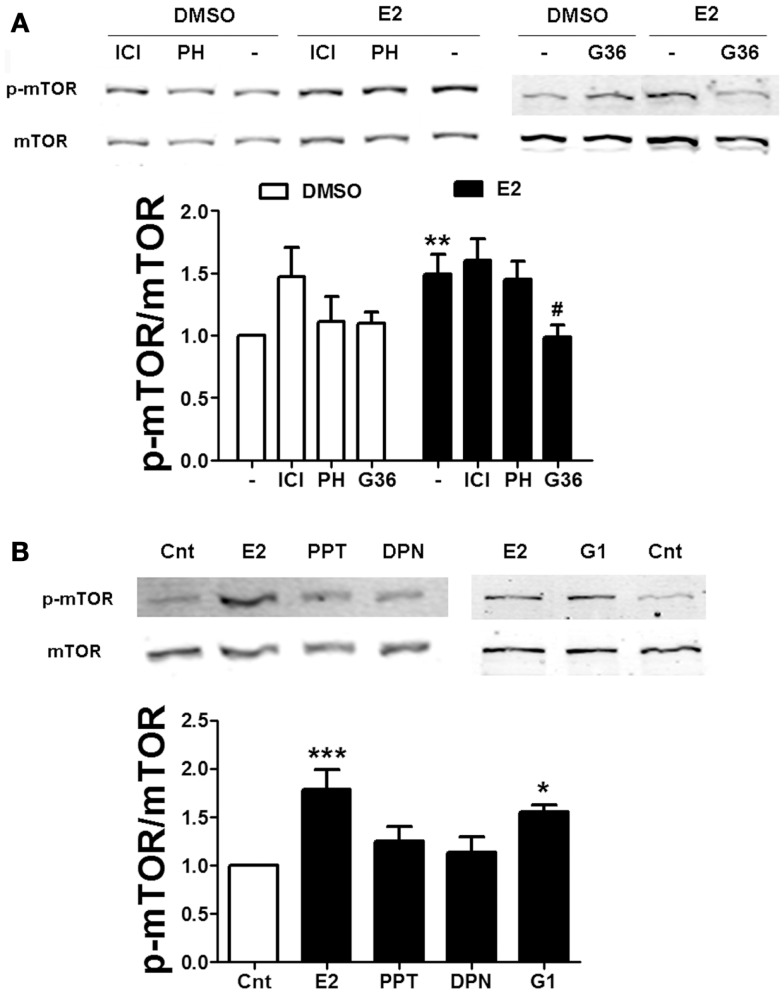
**Estradiol-induced mTOR phosphorylation involves GPR30 activation**. **(A)** Acute hippocampal slices were pre-treated with DMSO (Cnt), ICI 182780 (ICI, 1 μM), PHTPP (PH, 1 μM), or G36 (1 μM) for 30 min and then incubated with estradiol (E2, 10 nM) for additional 30 min. At the end of treatments, slices were homogenized and processed for western blot. Data are presented as the ratio (fold of control) of p-mTOR over total mTOR (*N* = 4–10). **(B)** Acute hippocampal slices were treated with estradiol (E2, 10 nM), PPT (100 nM), DPN (10 nM), or G1 (100 nM) and homogenized and processed for western blot. Data are presented as the ratio (fold of control) of p-mTOR over total mTOR (*N* = 4–6); *p < 0.05, ***p < 0.001, as compared to control (DMSO) (one- or two-way ANOVA).

To further examine the mechanism by which estradiol activates mTOR, we used specific agonists and antagonists of the two major ER isoforms, ERα and ERβ. Neither the non-specific ER antagonist ICI 182780 (ICI, 1 μM) nor the specific ERβ antagonist PHTPP (1 μM) affected estradiol-induced mTOR phosphorylation (Figure 4A). Consistent with this result, application of the ERα and ERβ specific agonists PPT (100 nM) or DPN (10 nM), respectively, did not stimulate mTOR activity (Figure 4B), suggesting that the effects of estradiol on the mTOR pathway do not involve activation of classical ERs. Some of the non-genomic effects of estradiol have been show to be mediated by the GPR30 (33). Thus, we tested the effects of a novel and very specific GRP30 antagonist, G36 (1 μM) (27), on estradiol-induced mTOR activation. This compound completely suppressed estradiol-elicited mTOR activation without affecting its basal levels (Figure 4A). In addition, treatment with the GPR30 agonist G1 (100 nM) enhanced mTOR phosphorylation to a similar extent as estradiol (Figure 4B). These results confirm that stimulation of the mTOR pathway by estradiol involves GPR30 activation. Interestingly, treatment with ICI alone significantly enhanced basal mTOR activity (Figure 4A). As will be discussed below, this effect might be related to its agonist activity at GPR30.

### Estradiol-induced actin polymerization involves ROCK, PAK, and ER activation

**Figure 5:**
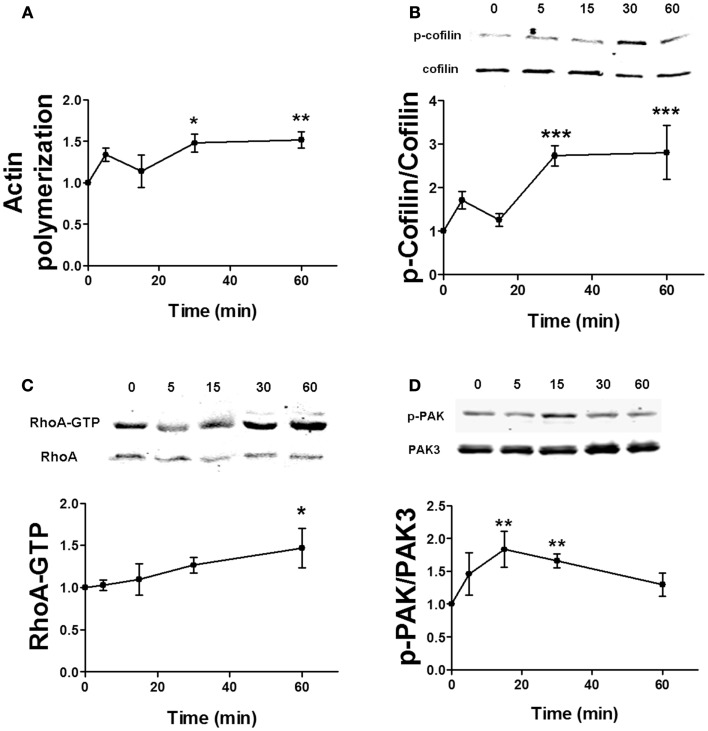
**Time course for estradiol-induced stimulation of actin polymerization**. **(A)** Acute hippocampal slices were treated with estradiol (10 nM) for the indicated periods of times, and were fixed, homogenized, and labeled with Phalloidin-TRITC (15–30 nM). Data are presented as fluorescence values with respect to basal fluorescence (fold of control). **(B–D)** Acute hippocampal slices were treated with estradiol (10 nM) for the indicated periods of times. After treatments, samples were homogenized and either processed for western blot **(B,D)** or pulled-down with RBD-bound agarose beads and then processed for western blot **(C)**. Data are presented as the ratio (fold of control) of: **(B)** phospho-cofilin (p-cofilin) over total cofilin (*N* = 4–8), **(C)** RhoA-GTP over total RhoA (*N* = 14–16), **(D)** phospho-PAK (p-PAK) over total PAK3 (*N* = 2–8); *p < 0.05, **p < 0.01, ***p < 0.001, as compared with control (0 min) (one-way ANOVA).

Estrogen has been shown to rapidly stimulate actin cytoskeleton dynamics via the RhoA/ROCK/cofilin pathway (6). It can also activate Rac/PAK signaling in neurons (14), but the role of this pathway in cofilin phosphorylation and actin polymerization has not yet been directly determined. Similarly, the time dependency for estradiol regulation of these pathways is uncertain. Thus, we first studied the temporal profile of the effects of estradiol on actin polymerization and on cofilin, RhoA, and PAK activation in hippocampal slices. Estradiol enhanced both actin polymerization and cofilin phosphorylation within 5 min, although these effects were not statistically significant. However, a significant activation of both actin polymerization and cofilin phosphorylation was observed 30 and 60 min after estradiol application (Figures 5A,B). Estradiol also increased RhoA and PAK activities although with different temporal patterns; the stimulatory effect of estradiol on RhoA activity was significant only at 60 min (Figure 5C), whereas PAK phosphorylation increased within 15–30 min and then returned to basal levels by 60 min (Figure 5D).

**Figure 6:**
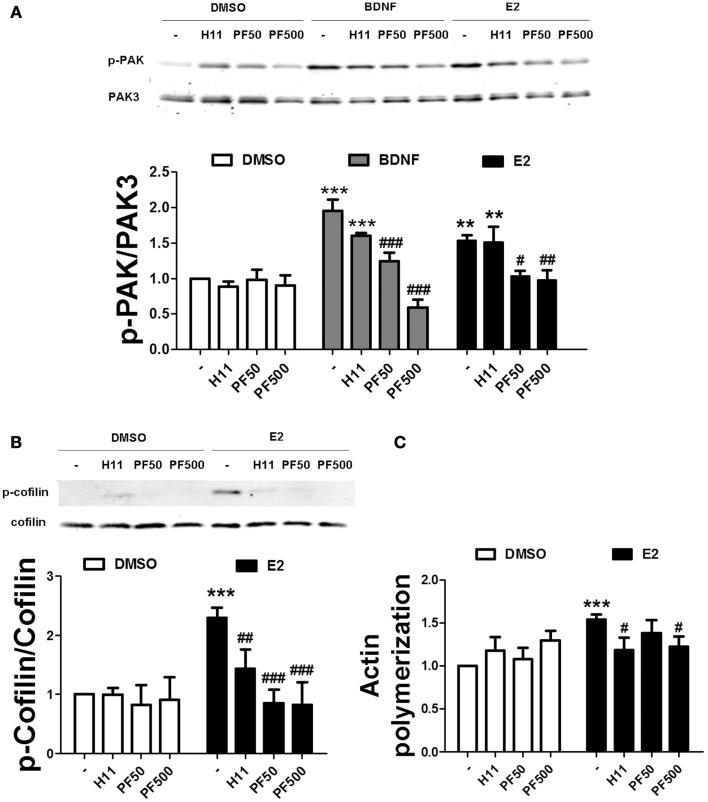
**Estradiol-induced actin polymerization requires ROCK and PAK activities**. **(A,B)** Acute hippocampal slices were pre-treated with DMSO (Cnt), H1152 (H11, 200 nM), or PF-3758309 (PF, 50–500 nM) for 30 min and then incubated with estradiol (E2, 10 nM) or BDNF (100 ng/ml) for additional 30 min. At the end of treatments, slices were homogenized and processed for western blot. Data are presented as the ratio (fold of control) of: **(A)** p-PAK over total PAK3 (*N* = 3–5), **(B)** p-cofilin over total cofilin (*N* = 3–10). **(C)** Treatments were performed as described above (for E2 the incubation time was 60 min) and slices were fixed, homogenized and labeled with Phalloidin-TRITC (15–30 nM). Data are presented as fluorescence values (fold of control) and are means ± SEM of four to eight independent experiments; **p < 0.01, ***p < 0.001, as compared to control (DMSO); ^##^*p* < 0.01, ^###^*p* < 0.001, as compared to E2 or BDNF alone (two-way ANOVA).

To examine the relative contribution of ROCK and PAK on estradiol-induced actin polymerization, we pre-treated hippocampal slices either with H1152 or PF-3758309, potent and specific inhibitors of ROCK and PAK, respectively. PF-3758309 has about a 10-fold higher selectivity for PAK1/4 (*K*_i_ values of 14/19 nM, respectively), as compared to PAK2/3 (*K*_i_ values of 190/99 nM, respectively) (34). Thus, we selected two different PF-3758309 concentrations (50 and 500 nM) in order to discriminate between PAK isoforms. We first verified the specificity of these inhibitors on BDNF- and estradiol-induced PAK phosphorylation by using an antibody that recognizes the activated forms of PAK1/2/3 isoforms. PF-3758309 blocked PAK activation caused by BDNF in a dose-dependent manner, although the effect was completely suppressed at the highest concentration only. In contrast, PF-3758309 totally inhibited estradiol-induced PAK activation at all the concentrations tested (Figure 6A) suggesting that BDNF and estradiol activate different PAK isoforms. As expected, treatment with H1152 did not affect the increase in PAK activity caused by either BDNF or estradiol (Figure 6A). Interestingly, both H1152 and PF-3758309 prevented the increase in cofilin phosphorylation and in actin polymerization induced by estradiol (Figures 6B,C), indicating that both pathways are involved in cofilin-dependent regulation of actin cytoskeleton by estrogen.

**Figure 7:**
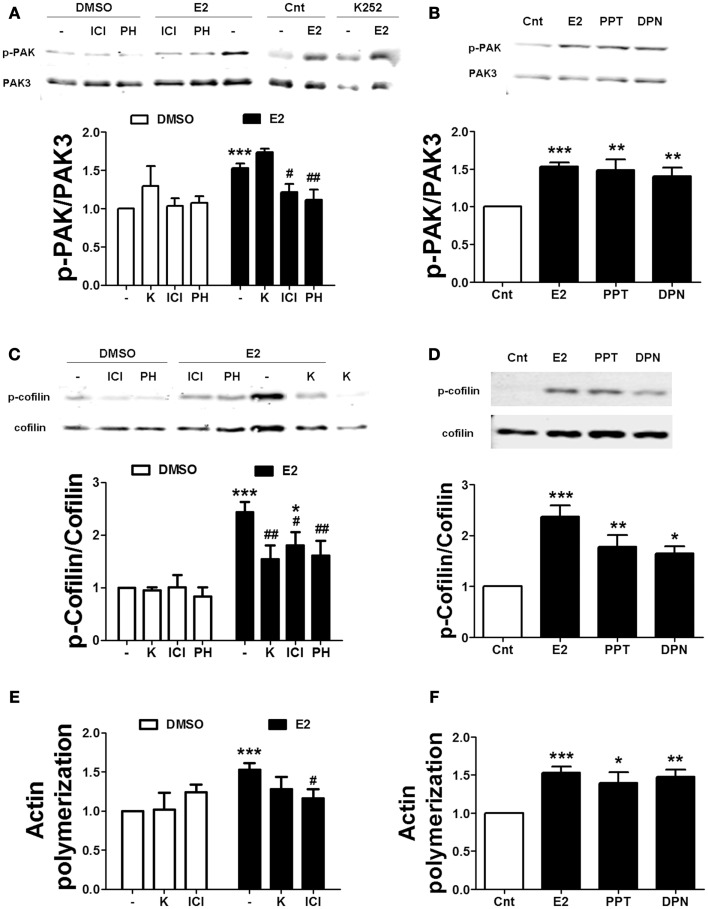
**Estradiol-induced actin polymerization involves classical ER activation**. **(A–D)** Acute hippocampal slices were pre-treated with DMSO (Cnt), ICI 182780 (ICI, 1 μM), PHTPP (PH, 1 μM), or K252 (K, 1 μM) for 30 min and then incubated with estradiol (E2, 10 nM), PPT (100 nM), or DPN (10 nM) for additional 30 min. At the end of treatments, slices were homogenized and processed for western blot. Data are presented as the ratio (fold of control) of **(A,B)** p-PAK over total PAK3 (*N* = 3–7), **(C,D)** p-cofilin over total cofilin (*N* = 3–11). **(E,F)** Treatments were performed as described above (for E2 the incubation time was 60 min) and then slices were fixed, homogenized and labeled with Phalloidin-TRITC (15–30 nM). Data are presented as fluorescence values (fold of control) and are means ± SEM of three to seven independent experiments; *p < 0.05, **p < 0.01, ***p < 0.001, as compared to control (DMSO); ^#^*p* < 0.05 ^##^*p* < 0.01, as compared to E2 alone (one- or two-way ANOVA).

We also tested the effects of ER and TrkB receptor antagonists on estrogen-mediated actin reorganization. Treatment with either ICI or PHTPP blocked the effects of estradiol on PAK and cofilin phosphorylation. In contrast, K252a failed to inhibit estradiol-induced PAK activation, but it prevented the increase in cofilin phosphorylation caused by the hormone (Figures 7A,C). Consistent with the above data, ICI suppressed estradiol-induced actin polymerization. K252a reduced the stimulatory effect of estradiol on actin polymerization, although the effect was not significant (Figure 7E). To further confirm the involvement of ERs in the cytoskeletal changes elicited by estrogen, we tested the effects of the specific ER agonists on the same endpoints. Both PPT and DPN significantly increased PAK and cofilin phosphorylation (Figures 7B,D). Similarly, the two agonists stimulated actin polymerization to a similar extent as estradiol (Figure 7F). These results suggest that both ERα and ERβ are involved in estradiol regulation of the actin cytoskeleton.

### Estradiol-induced mTOR activation requires actin polymerization and PAK activation

We next evaluated the relationship between protein synthesis and cytoskeletal reorganization after exposure to estrogen in order to determine whether these two events are independent. To address this question, we determined the effects of protein synthesis inhibitors on estradiol-elicited actin polymerization in hippocampal slices. Neither 25 μM cycloheximide nor 1 μM rapamycin blocked estradiol-induced actin polymerization (Figure S1 in Supplementary Material). Estrogen-mediated stimulation of dendritic protein translation has been shown to be ERK-dependent (22). However, under our experimental conditions, treatment with the ERK kinase inhibitor U0126 (5 μM) failed to prevent estradiol-induced actin polymerization (Figure S1 in Supplementary Material). These results indicate that protein synthesis is not required for estradiol-mediated regulation of cytoskeletal dynamics.

**Figure 8:**
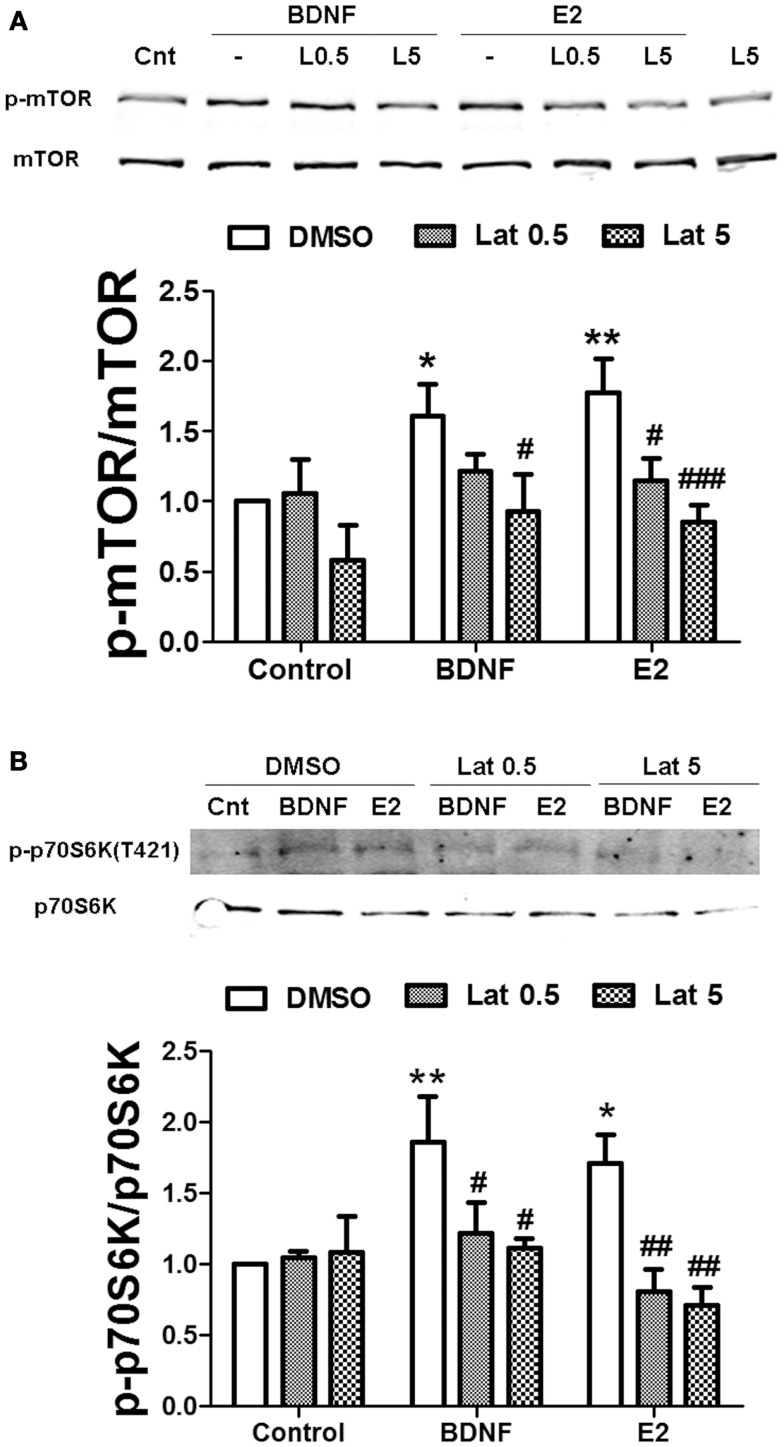
**Estradiol-induced mTOR-dependent and mTOR–independent protein translation requires actin polymerization**. Acute hippocampal slices were pre-treated with DMSO (Cnt) or Latruncullin A (Lat, 0.5–5 μM) for 30 min and then incubated with estradiol (E2, 10 nM) or BDNF (BD, 100 ng/ml) for an additional 30 min. At the end of treatments, slices were homogenized and processed for western blot. Data are presented as the ratio (fold of control) of: **(A)** p-mTOR over total mTOR (*N* = 4–5), **(B)** p-p70S6K (T421/S424) over total p70S6K (*N* = 4); *p < 0.05, **p < 0.01, as compared to control (DMSO); ^#^*p* < 0.05, ^##^*p* < 0.01, ^###^*p* < 0.001, as compared to E2 or BDNF alone (two-way ANOVA).

We then determined whether actin polymerization was necessary for estradiol-mediated mTOR stimulation. Thus, we pre-treated hippocampal slices with Latrunculin A (Lat A), an agent that sequesters actin monomers, thereby preventing filamentous actin (F-actin) assembly. Two concentrations of Lat A were used (0.5 and 5 μM) based on the different sensitivities of F-actin to this drug depending on its subcellular location; low concentrations of Lat A specifically target actin filaments within the dendritic shaft, while higher concentrations are required to affect dendritic spine F-actin (31). Treatment with 0.5 μM Lat A alone had no effect on basal mTOR activity. In contrast, the depolymerizing agent slightly reduced mTOR phosphorylation at 5 μM, although the effect did not reach statistical significance. Interestingly, Lat A caused a concentration-dependent reduction of estradiol-mediated mTOR phosphorylation in hippocampal slices. Under the same conditions, BDNF-induced mTOR activation was also impaired, but the effect was only significant at the highest concentration of Lat A tested (Figure 8A). Similarly, BDNF- and estradiol-induced phosphorylation of p70S6K both at Thr389 (Figure S2A in Supplementary Material) and at Thr421/Ser424 (Figure 8B) was totally suppressed by Lat A at all the concentrations tested. In contrast, the increase in Akt phosphorylation caused by BDNF and estradiol was significantly reduced by Lat A at the highest concentration only (Figure S2B in Supplementary Material). These results suggest that actin polymerization is required for mTOR-dependent protein translation elicited by BDNF and estradiol.

**Figure 9:**
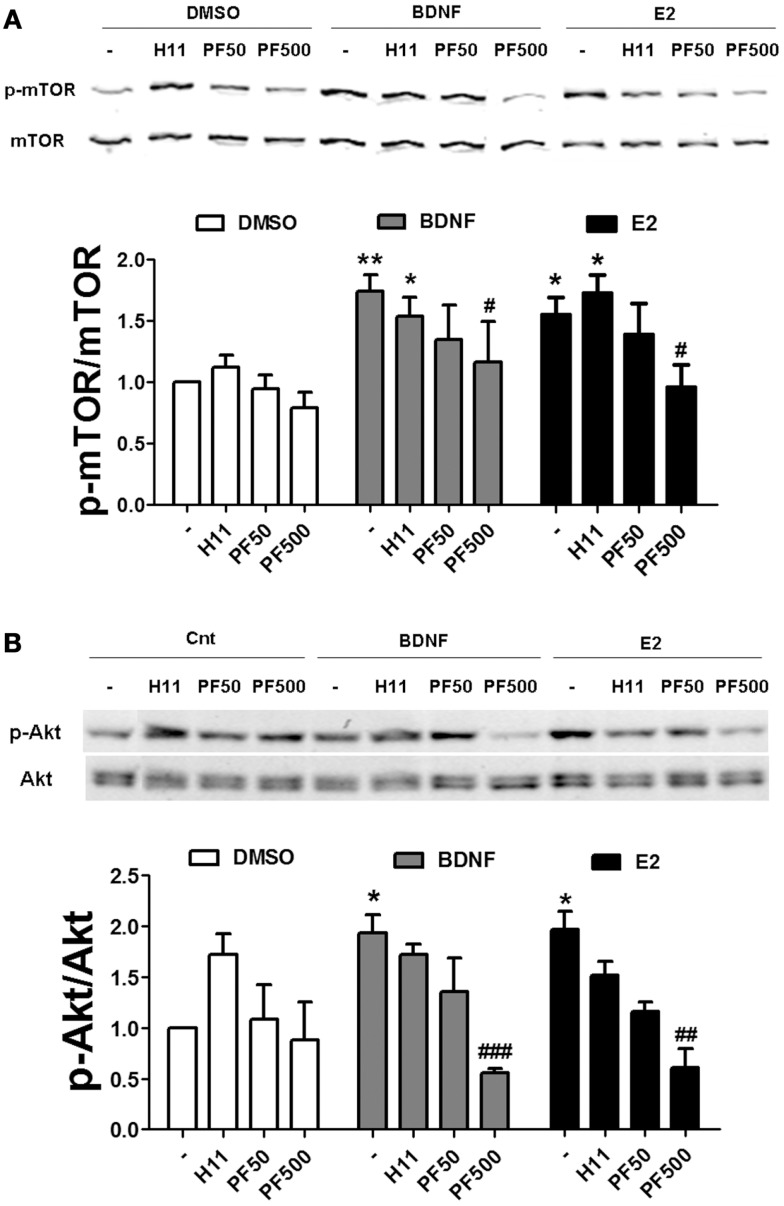
**Estradiol-induced mTOR signaling requires PAK activation**. **(A)** Acute hippocampal slices were pre-treated with DMSO (Cnt), H1152 (H11, 200 nM) or PF-03758309 (PF, 50–500 nM) for 30 min and then incubated with estradiol (E2, 10 nM) or BDNF (100 ng/ml) for an additional 30 min. At the end of treatments, slices were homogenized and processed for western blot. Data are presented as the ratio (fold of control) of: **(A)** p-mTOR over total mTOR (*N* = 3–13),**(B)** p-mTOR over total mTOR (*N* = 3–4); *p < 0.05, **p < 0.01, as compared to control (DMSO); ^#^*p* < 0.05, ^##^*p* < 0.01, ^###^*p* < 0.001, as compared to E2 or BDNF alone (two-way ANOVA).

If actin polymerization regulates estradiol-mediated mTOR activation/phosphorylation, it could be due to an activity-dependent event triggered by activation of classical pathways (i.e., RhoA/ROCK and/or Rac/PAK) or be due to a structural requirement linking activated Akt to mTOR. We addressed this question by determining the effects of specific ROCK and PAK inhibitors on estradiol- and BDNF-induced mTOR and Akt activation. Treatment with H1152 did not affect mTOR phosphorylation stimulated by BDNF or estradiol in hippocampal slices. In contrast, 500 nM (but not 50 nM) PF-3758309 completely blocked BDNF- and estradiol-induced mTOR activation (Figure 9A). Likewise, the increase in phospho-Akt caused by BDNF and estradiol was prevented by PF-3758309 only at the highest concentration tested. Surprisingly, treatment with H1152 alone enhanced Akt phosphorylation but the effect was not statistically significant (Figure 9B). This result is in agreement with previous studies linking ROCK inhibition to stimulation of Akt signaling pathway in neuronal cells (35, 36). Overall, these findings suggest that activation of PAK2/3 signaling by estradiol and BDNF regulates mTOR signaling, possibly through stimulation of actin polymerization.

## Discussion

**Figure 10:**
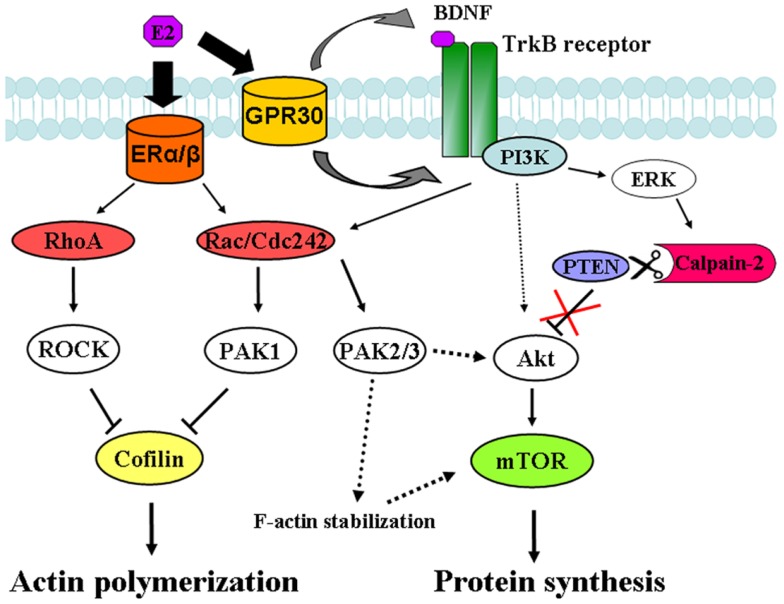
**Signaling pathways involved in estradiol regulation of protein synthesis and actin polymerization in hippocampal neurons**. Estradiol action on classical ERα and ERβ activates both RhoA/ROCK and Rac/Cdc42/PAK1 signaling pathways leading to cofilin inactivation and stimulation of actin polymerization. On the other hand, estradiol action on non-classical ER, GPR30 induces TrkB receptor activation, which triggers ERK-dependent calpain-2 activation leading to PTEN cleavage and subsequent stimulation of the Akt/mTOR-mediated protein synthesis. In addition, activation of PAK2/3 signaling by BDNF and estradiol contributes to Akt stimulation and/or supports F-actin stability, which is necessary for mTOR activation.

In the present study, we found that estradiol stimulates mTOR in acute hippocampal slices through a signaling pathway involving TrkB receptor activation, ERK phosphorylation, and calpain activity. Specific inhibitors of each of these enzymes abrogated the increase in mTOR phosphorylation elicited by estradiol. These results are consistent with the recently identified mechanism linking BDNF-induced activation of the PI3K/Akt signaling cascade and stimulation of mTOR-dependent protein synthesis through calpain-2-mediated PTEN truncation (24) (see schematic in Figure 10). In a previous study, we reported that BDNF and epidermal growth factor (EGF) specifically activated calpain-2 within dendritic spines through ERK-mediated phoshorylation (28). Similarly, estradiol rapidly activates calpain in hippocampal neurons and this process has been implicated in estrogen modulation of synaptic plasticity (8). The time course for estradiol regulation of ERK, Akt and mTOR phosphorylation as well as calpain activation, and PTEN degradation is in good agreement with that of BDNF-mediated regulation of the same sequence of events observed in our previous study (24). Thus, estradiol enhanced ERK activity within 5 min of application both in hippocampal slices and in cortical synaptoneurosomes, and this effect occurred in parallel to stimulation of calpain activity, as determined by the increase in SBDP. This was followed by a decrease in PTEN levels along with an increase in Akt and mTOR phosphorylation 15 min after estradiol application. The activation of all these signaling proteins was transient, except for that of mTOR, which persisted for at least 1 h after estradiol treatment. This time course is thus in good agreement with our previous study in which we showed that calpain, in addition to PTEN, causes the degradation of two of the negative regulators upstream of mTOR (and downstream of Akt), namely hamartin and tuberin (24).

Previous studies have shown that the stimulation of dendritic protein translation by estradiol involves ERK and Akt activities (21, 22). While it is widely accepted that Akt regulates mTOR-mediated protein synthesis, it is still not clear how ERK signaling stimulates the translation machinery, as several reports indicate that ERK activation can trigger both mTOR-dependent and independent translation mechanisms (37–39). In our study, both estradiol- and BDNF-induced mTOR phosphorylation were blocked by a specific ERK inhibitor, a result consistent with the involvement of ERK in calpain-2 activation and subsequent stimulation of the Akt/mTOR pathway. These results are also in good agreement with our previous finding showing that estradiol-induced Akt activation in synaptoneurosomes was suppressed by two different ERK kinase inhibitors (25). In addition, ERK activation has been reported to mediate mTOR-dependent enhancement of object recognition produced by estradiol injection into the dorsal hippocampus (23). These results do not exclude the possibility that ERK can also modulate protein synthesis upstream of mTOR, through the regulation of tuberin (37), or downstream of it, by phosphorylation of p70S6K at Thr421/Ser424 (39). Indeed, the fact that CI-III did not block estradiol-induced p70S6K phosphorylation at these sites suggests an additional mTOR-independent regulation of protein translation by the hormone, as was previously observed for BDNF (24).

Recent studies have focused on the importance of estradiol and BDNF cross-talk signaling in the regulation of synaptic function (3, 4, 40), but the potential mechanisms involved are still largely unknown. Here, we provide evidence that estradiol and BDNF mediate some of their rapid actions in hippocampus through common signaling cascades. Moreover, our findings strongly suggest that estrogen regulation of mTOR-dependent protein translation is mediated by BDNF, since the TrkB receptor antagonist K252a completely suppressed estradiol-induced mTOR activation. In this regard, estrogen rapid and reversibly enhances TrkB receptor phosphorylation in dendritic spines of CA1 pyramidal cells (3). In addition, estradiol stimulates synaptogenesis in dentate gyrus through PKA-mediated BDNF release (41), although the effects were observed several hours after estradiol application and at a very high, non-physiological concentration of the hormone. Yet, the estrogenic effects on mTOR activity might be caused as well by a non-genomic and BDNF-dependent mechanism considering that estradiol is able to rapidly stimulate PKA activation in hippocampal neurons (42). Alternatively, estradiol could directly transactivate TrkB receptor via intracellular signaling cascades, as was found for other tyrosine kinase receptors, such as those for EGF and insulin-like growing factor (33, 43). Future work will be directed at testing these hypotheses.

The stimulation of the mTOR pathway by estrogen did not require activation of classical ERs, as both the agonists, PPT and DPN, failed to mimic the effect of the hormone on phospho-mTOR levels, and the antagonists, ICI and PHTPP, to inhibit it. Although some studies have shown that estradiol stimulates Akt/mTOR signaling via classical ER activation (44, 45), the effects were observed several days after hormone exposure suggesting different mechanisms for short- vs. long-term estrogenic actions. It is worth noting that stimulation of BDNF release by estradiol does not require classical ER activation (41), reinforcing the hypothesis of a BDNF-mediated, but ER-independent, mechanism for estradiol regulation of the mTOR pathway. Our results indicate that estradiol-induced mTOR phosphorylation is mediated by GPR30, as the effect was blocked by the specific GPR30 antagonist G36 and mimicked by its agonist, G1. Interestingly, ICI stimulated mTOR activity to a similar extent as estradiol and estradiol and ICI did not have additive effects when applied together. This result further supports the notion that the effects of estradiol on mTOR involve GPR30, as ICI has been shown to act as an agonist for this receptor (37). Growing evidence points to GPR30 as an important mediator of some of the non-genomic actions of estrogen (33, 46). This receptor has been recently identified in hippocampal dendritic spines in association with the post-synaptic density (47), suggesting a potential involvement of GPR30 in estrogen modulation of synaptic plasticity. Our findings indicate a novel role for GPR30 as a regulator of mTOR signaling pathway, which is a key component of estrogen modulation of hippocampal-dependent memory consolidation (23). In addition, GPR30 has been reported to regulate neuritogenesis in developing hippocampal neurons via PI3K/Akt activation (48). It would be interesting to test whether this effect requires mTOR-mediated protein synthesis and/or TrkB receptor activity, considering the prominent role of BDNF in axonal growth.

Classical ER activation was required for PAK and cofilin phosphorylation, as well as for actin polymerization elicited by estradiol. Furthermore, both ERα and ERβ agonists stimulated actin signaling and cytoskeletal reorganization. In a previous study (6), only an ERβ agonist was able to reproduce estradiol-mediated facilitation of LTP, which was shown to depend on actin cytoskeletal dynamics. However, the effects of specific ER ligands were not tested on the signaling proteins involved in the regulation of actin cytoskeleton. Noteworthy, rapid phosphorylation of PAK1 by estradiol in hypothalamus has been attributed to non-classical ERα signaling (15). In addition, several studies have implicated ERα in the positive effects of estradiol on synaptogenesis (7, 49), while the use of knock-out mice indicated a major role for ERβ in LTP and spatial memory (50). Despite this, recent work indicates that both DPN and PPT enhance novel object recognition and object placement memory (51, 52), suggesting that both ER isoforms contribute to the improvement in cognitive performance produced by estrogen.

The reorganization of the actin cytoskeleton plays a major role in the structural and functional changes that occur in dendritic spines during LTP (9, 10). Estrogen has been reported to rapidly stimulate actin polymerization in the CA1 area of hippocampus and this effect has been linked to the increase in AMPA receptor-mediated post-synaptic responses induced by the hormone (6). Kramar and collaborators found that 20 min of exposure to 1 nM estradiol activates RhoA but not Rac, Cdc42, or PAK. In addition, they showed that the ROCK inhibitor H1152 blocked the effects of estrogen on synaptic transmission. Here, we report that estradiol stimulates actin cytoskeletal dynamics via both RhoA/ROCK and PAK signaling cascades (Figure 10), as estradiol increased both RhoA and PAK activation and specific inhibitors of either ROCK or PAK suppressed estradiol-induced cofilin phosphorylation and actin polymerization. The differences between their studies and ours could be due to different concentrations of estradiol (1 vs. 10 nM) and/or different ages of the animals used (4–5 vs. 8–12 week-old rats). In support of the latest possibility, a lack of estrogen responsiveness (i.e., on Akt activity) was found in pre-pubertal (postnatal day 30), as compared to post-pubertal (postnatal day 60) rats (53). Therefore, it is possible that activation of Rac/PAK, as opposed to RhoA/ROCK, has not yet completely developed in adolescent rats. It is also widely acknowledged that estradiol exhibits a biphasic dose–response for some of its non-genomic and genomic actions (54, 55). In particular, estradiol was found to increase Rac/Cdc42 activity at the same concentration used in our study (12, 13). In any event, the lowest concentration of PF-3758309 (which presumably blocks PAK1/4 but not PAK2/3) was sufficient to completely abrogate the effects of estradiol on PAK and cofilin phosphorylation. Since the phospho-PAK antibody we used only recognizes activated PAK1/2/3 isoforms, our results indicate that PAK1 activity is required for the increase in cofilin phophorylation induced by estradiol. However, we cannot rule out the involvement of PAK4 in the cytoskeletal rearrangement elicited by the hormone, given that it can also stimulate LIMK1 activity, resulting in cofilin phosphorylation and inactivation (17). It is worth noting that PF03758309 totally suppressed the effects of estradiol on actin polymerization and mTOR phosphorylation only at the highest concentration tested, suggesting a possible role for PAK2/3 in estradiol regulation of actin cytoskeletal dynamics as well as of mTOR signaling.

Interestingly, actin polymerization/stabilization was required for estradiol-induced stimulation of protein translation signaling, as the depolymerizing agent Lat A suppressed BDNF- and estradiol-induced mTOR and p70S6K phosphorylation. To the best of our knowledge, this is the first study showing that rapid mTOR activation requires F-actin treadmilling. In contrast, protein synthesis was not required for estradiol-induced actin polymerization. Furthermore, we provided evidence that PAK activity was also necessary for mTOR activation, since the PAK specific inhibitor (at the highest concentration used only) but not the ROCK inhibitor blocked Akt and mTOR phosphorylation induced by both BDNF and estradiol. Although some studies have linked PAK1/2 to the Akt signaling pathway independently of its regulatory action on actin filaments (56–58), our results suggest that blockade of PAK2/3 activity may prevent activity-induced stabilization of the actin cytoskeleton within dendrites and hence stimulation of mTOR-dependent protein synthesis (Figure 10). It has been previously reported that translocation of the translation machinery to dendritic spines is dependent on actin cytoskeletal dynamics and represent a key event in activity-dependent synaptic plasticity (31, 59). In addition, PAK has been previously implicated in insulin-mediated gene expression through the regulation of the actin cytoskeleton (60). In this study (60), actin depolymerizing agents did not affect nuclear mTOR activation induced by insulin, but reduced its activation in the cytoplasm, which indicates the specificity of these drugs to target specific populations of actin filaments. In this regard, low concentrations of Lat A have been reported to specifically target actin filaments in dendritic shafts (where protein translation is generally thought to take place) but not in dendritic spines (31). In our hands, a similar low concentration of Lat A blocked mTOR and p70S6K (but not Akt) activation by BDNF or estradiol without affecting basal levels of phospho-mTOR or phospho-p70S6K, excluding the possibility that the effect was the result of a non-specific disruption of the whole actin cytoskeleton architecture. These findings, together with those obtained with the PAK inhibitor, support the idea that the regulation of mTOR-dependent protein synthesis by the actin cytoskeleton is not merely a constitutive process but an activity-driven phenomenon. Thus, actin filaments probably not only function as a structural component necessary to recruit the mTOR complex and associated translation factors to the plasma membrane, but might also actively participate in the regulation of their transport and activity.

In summary, the results present here shed new light on the molecular mechanisms involved in estrogen modulation of hippocampal synaptic plasticity, and underscore the roles of distinct signaling pathways in mediating its effects on protein synthesis and actin cytoskeleton reorganization.

## Author Contributions

Victor Briz performed all the experiments, prepared the figures, and wrote the manuscript. Michel Baudry directed the research and wrote the manuscript.

## Conflict of Interest Statement

The authors declare that the research was conducted in the absence of any commercial or financial relationships that could be construed as a potential conflict of interest.

## Supplementary Material

The Supplementary Material for this article can be found online at http://www.frontiersin.org/Journal/10.3389/fendo.2014.00022/abstract
