# Supplementary material for: Estrogen Regulates Protein Synthesis and Actin Polymerization in Hippocampal Neurons through Different Molecular Mechanisms
Source: Front Endocrinol (Lausanne). 2014 Feb 25;5:22. doi: 10.3389/fendo.2014.00022 (PMC3933789; doi:10.3389/fendo.2014.00022)
Supplement: Supplementary file 1 [file 79210_Baudry_DataSheet1.PDF]

SUPPLEMENTARY MATERIAL

Supplementary Figure 1

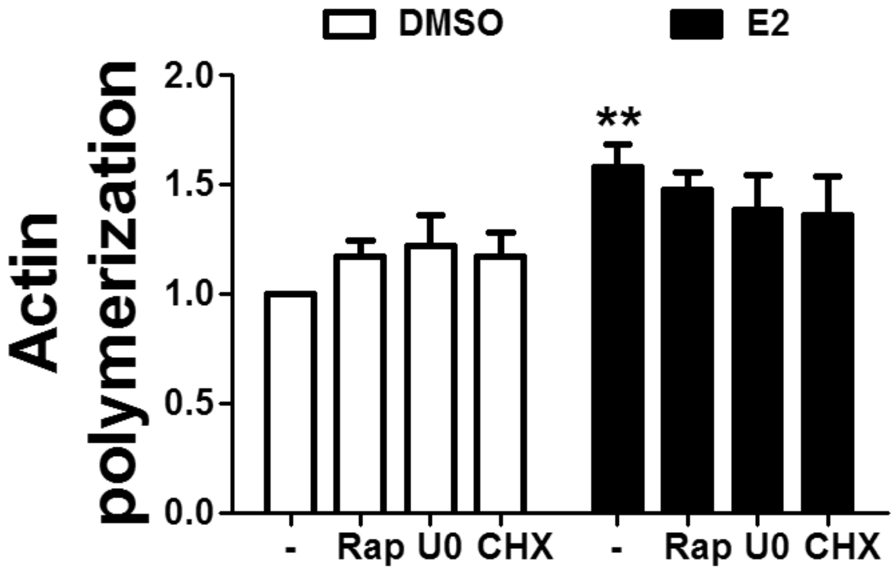

Supplementary Figure 1: Estradiol-induced actin polymerization does not require protein synthesis. Acute hippocampal slices were pre-treated with DMSO (Cnt), Rapamycin (Rap, 1 μM), U0126 (U0, 5 μM) or cycloheximide (CHX, 25 μM) for 30 min and then incubated with estradiol (E2, 10 nM) for an additional 60 min. After treatments, slices were fixed, homogenized and labelled with Phalloidin-TRITC (15-30 nM). Data are presented as fluorescence values (fold of control) and are means ± SEM of 4-5 independent experiments; ** $p<0.01$, as compared to control (DMSO) (two-way ANOVA).

Supplementary Figure 2

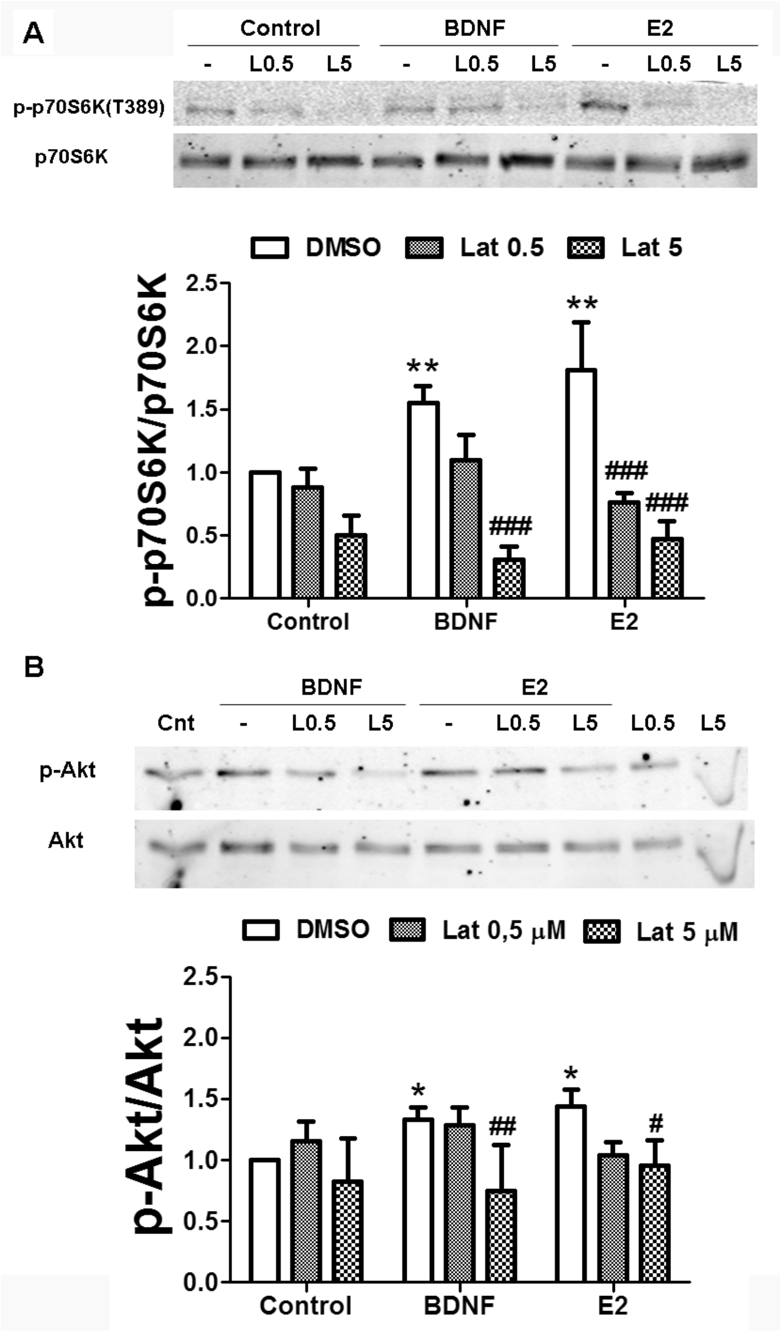

Supplementary Figure 2: Estradiol-induced mTOR signaling requires actin polymerization. Acute hippocampal slices were pre-treated with DMSO (Cnt) or Latruncullin A (Lat, 0.5-5 μM) for 30 min and then incubated with estradiol (E2, 10 nM) or BDNF (BD, 100 ng/ml) for additional 30 min. At the end of treatments, slices were homogenized and processed for western blot. Data are presented as the ratio (fold of control) of: **(A)** p-p70S6K (T389) over total p70S6K (N=3-7), **(B)** p-Akt over total Akt (N=4-8); * $p<0.05$, *** $p<0.001$, as compared to control (DMSO); # $p<0.05$, ## $p<0.01$, ### $p<0.001$, as compared to E2 or BDNF alone (two-way ANOVA).
